# Obacunone, a Promising Phytochemical Triterpenoid: Research Progress on Its Pharmacological Activity and Mechanism

**DOI:** 10.3390/molecules29081791

**Published:** 2024-04-15

**Authors:** Yuyang Zhou, Jifeng Gu, Jiahui Li, Huishan Zhang, Mei Wang, Yuanyuan Li, Tianming Wang, Jiajie Wang, Rong Shi

**Affiliations:** 1 Science and Technology Experimental Center, Shanghai University of Traditional Chinese Medicine, Shanghai 201203, China; yzhou78@uthsc.edu (Y.Z.); judyli987@163.com (J.L.); zhanghuishan0729@126.com (H.Z.); wangmei202300@163.com (M.W.); 18805864529@163.com (J.W.); 2 Department of Pharmacology, Addiction Science and Toxicology, University of Tennessee Health Science Center, College of Medicine, Memphis, TN 38163, USA; 3 Shanghai Key Laboratory of Bioactive Small Molecules, Fudan University, Shanghai 200032, China; jifeng.gu@fdeent.org; 4 Department of Pharmacy, Eye & ENT Hospital, Fudan University, Shanghai 200031, China; 5 Longhua Hospital, Shanghai University of Traditional Chinese Medicine, Shanghai 200032, China; 6 Department of Pharmacology, Shanghai University of Traditional Chinese Medicine, Shanghai 201203, China; yuanyuan60107@163.com (Y.L.); wtmtcm@126.com (T.W.); 7 Shuguang Hospital, Shanghai University of Traditional Chinese Medicine, Shanghai 200021, China

**Keywords:** obacunone, pharmacology, molecular mechanism, antiviral activity, limonoid

## Abstract

Obacunone, a natural triterpenoid, is an active component of the herbs *Dictamnus dasycarpus* Turcz. and *Phellodendron amurense* Rupr, and an indicator of the herbs’ quality. Owing to its multiple health benefits, several studies have investigated the multi-targeting potential action mechanisms of obacunone. To summarize recent developments on the pharmacological actions of obacunone and focus on the underlying molecular mechanisms and signaling networks, we searched PubMed, Europe PMC, Wiley Online Library, Web of Science, Google Scholar, Wanfang Medical Network, and China National Knowledge Infrastructure for articles published prior to March 2024. Existing research indicates obacunone has great potential to become a promising therapeutic option against tumors, fibrotic diseases, bone and cholesterol metabolism diseases, and infections of pathogenic microorganisms, among others. The paper contributes to providing up-to-date references for further research and clinical applications of obacunone.

## 1. Introduction

**Figure 1:**
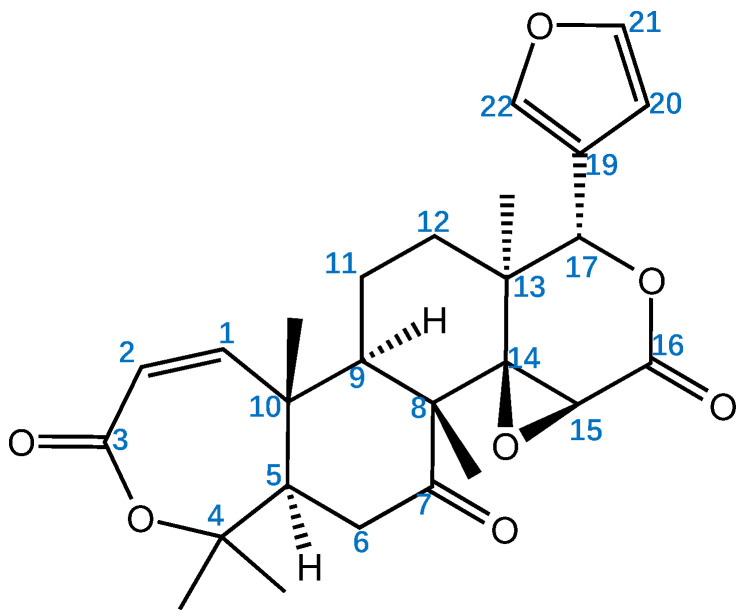
Structure of obacunone.

Obacunone (PubChem CID 119041), a member of the class of limonoids, is found in various herbs, especially in eastern Asia. Obacunone is a marker for assessing the qualities of the herbs *Dictamnus dasycarpus* Turcz. (Bai Xian Pi) and *Phellodendron amurense* Rupr (Guan Huang Bo) in the Chinese Pharmacopoeia and a primary active component of multiple traditional Chinese medicine, such as *Coptis chinensis* Franch. (Huang lian), *Euodia rutaecarpa* (Juss.) Benth. (Wu zhu yu), ShangKeHuangShui, Erchen decoction, Chaihu Shugan San, and the Huanglian Jiedu decoction (Table 1). As a member of the limonin family, obacunone is a highly oxidized secondary metabolite of tetracyclic triterpenoid plants with a basic framework structure of 4,4,8-trimethyl-17-furanyl steroids (Figure 1); oxygen functional groups are present at positions C-3, C-4, C-7, C-16, and C-17, and a furan ring is located on C17, which is a key functional group that facilitated various biological properties such as anticancer, anti-inflammation, and antiviral activities [1].

Obacunone has received increasing attention owing to its pharmacological properties. Zheng et al. have published a narrative review of the relevant research on obacunone [13]. However, due to the rapid progress in research, especially in the exploration of deeper mechanisms in anticancer, anti-inflammatory, anti-oxidative stress and anti-fibrosis activities, their effects on bone metabolism, the regulation of cholesterol metabolism, and actions against anti-pathogenic microorganisms, especially their potential efficacy against coronavirus disease 2019 (COVID-19) viruses, previous literature did not include new findings. Therefore, this review provides a more detailed and comprehensive updated summary of the pharmacological and pharmacokinetic progress of obacunone. The findings discussed in this review will expand our understanding of obacunone and help facilitate its development and clinical applications.

## 2. Methodology

A literature review was conducted in this study using the scientific search engines and databases PubMed, Europe PMC, Wiley Online Library, Web of Science, Google Scholar, Wanfang Medical Network (med.wanfangdata), China National Knowledge Infrastructure, DBpia, and Korea National Assembly Electronic Library. The search terms “Obacunone”, “Pharmacology”, “Toxicology”, “Cytotoxicity”, and “Pharmacokinetics” were employed to identify studies related to the pharmacological, toxicological, and pharmacokinetic properties of obacunone published prior to March 2024, including other relevant literature. A total of 106 peer-reviewed studies published in English, Chinese, and Korean journals, including some doctoral and master’s degree theses, were included in the analysis.

## 3. Pharmacological Effects of Obacunone

### 3.1. Antitumor Effects 

#### 3.1.1. Antitumor Activity In Vivo

The anticancer activity of obacunone is currently focused on its effect on colon cancer, and obacunone has demonstrated remarkable anti-colon cancer effects [14,15,16]. In a murine model of colon cancer induced with azoxymethane (AOM)/dextran sodium sulfate (DSS), intragastric administration of obacunone (50 mg/kg) resulted in a significant reduction in bloody diarrhea, inflammatory responses, and the number of positive proliferating cell nuclear antigens in the colon; a decrease in the incidence, size, and diversity of tumors; and a substantial increase in colon length [16]. Mill et al. investigated the anti-tumor activity of three homologous compounds of limonin, namely obacunone, ichangensin, and deoxyglimonin, in a hamster cheek pouch model induced with 7,12-dimethylbenz[*a*]anthracene. The authors found that obacunone and deoxyglimonin had inhibitory effects on cancer with similar efficacy, while ichangensin had no significant effects [17], indicating the importance of A-rings in anti-tumor activity [1].

#### 3.1.2. Antitumor Activity In Vitro

##### Effects on Gastrointestinal Tumors: Colon, Liver, and Pancreatic Cancer Cells

In in vitro experiments, obanone inhibited the proliferation of human colon cancer Caco-2 [16,18], HT-29 [16], SW480 [16,19], and HCT-116 cells [16] in a dose-dependent manner, decreased the growth of cancer cells in the G1 and G2 phases, reduced the total number of cells in the S-phase, and induced endogenous apoptosis without affecting the growth of normal cells [16]. Moreover, when combined with other anticancer drugs, obacunone has been shown to enhance the effects of the drugs. For example, obacunone enhanced the effects of camptothecin (specifically proliferation inhibition and apoptosis activation [25 μM]) in human colon cancer cells [19] and reversed P-glycoprotein (P-gp)-induced multidrug resistance in drug-resistant human colon cancer HCT15 cells, thus significantly enhancing the cytotoxicity of paclitaxel [20].

In addition, obacunone may have potential antihepatocellular and antipancreatic cancer effects. Indeed, obacunone has been reported to reduce the viability of hepatocellular carcinoma HepG2 cells dose-dependently and decrease their proliferation by affecting cell membrane permeability, nuclear intensity, and reactive oxygen species (ROS) concentrations [21]. In vitro, obacunone showed better docking scores with SRC kinase, a primary target of the antiliver cancer effect of *Evodiae fructus*, and was linked to residues via hydrogen and hydrophobic bonds according to the 2D/3D-QSAR pharmacophore model and the stepwise multiple linear regression approach [4]. Obacunone also inhibited the proliferation of pancreatic cancer Panc-28 cells and activated Caspase-3 by inducing cytochrome C release in a time- and dose-dependent manner, promoting the apoptosis of Panc-28 cells [22].

##### Effects on Prostate Cancer Cells

Obacunone plays a potential role in inhibiting prostate cancer [23]. Obacunone inhibited the cellular Akt signaling pathway; increased cancer cell DNA fragmentation, caspase-3 activity, and cytoplasmic cytochrome C levels; inhibited cell proliferation; dose-dependently and time-dependently induced apoptosis, significantly decreasing inflammatory and cancer cell marker levels in LNCaP cells. Notably, obacunone specifically killed prostate cancer cells without harming normal prostate RWPE-1 cells [24]. Similar to its effect on LNCaP cells, obacunone inhibited the proliferation of 22RV1 prostate cancer cells, and the mechanism underlying this effect may be related to niacin and nicotinamide, ascorbate and aldehyde, tryptophan, phenylalanine, and galactose metabolism [25].

##### Effects on Breast Cancer Cells

Obacunone exhibited significant cytotoxicity against human breast cancer MDA-MB-231 and MCF-7 cells. Mechanistic studies revealed that obacunone may exert potent cytotoxic effects on MCF-7 cells by inhibiting aromatase activity from affecting the estrogen receptor (ER). Its cytotoxicity to MDA-MB-231 cells was significantly attenuated owing to the mutations in p53 and ER genes [26].

##### Effects on Female Reproductive System Cancer Cells

Although evidence of the direct effect of obacunone on the female reproductive system cancer is lacking, obacunone has been shown to reverse cellular resistance when used in combination with typical chemotherapeutic agents (such as vincristine) [18,27]. Similar to that in the resistance in human colon cancer HCT15 cells, obacunone inhibited P-gp activity in human uterine sarcoma MES-SA/DX5 cells and significantly enhanced the cytotoxicity of paclitaxel [20]. Obacunone increased the cytotoxicity of vincristine against KB-3-1 and KB-V1 cells, mutants of human cervical cancer HeLa cells, with KB-V1 cells being particularly resistant, by 4-fold and 16-fold, respectively, which may not be related to its effect on P-gp [28].

##### Effects on Neuroblastoma Cells

Obacunone treatment of human neuroblastoma SH-SY5Y cells significantly inhibited cell growth. Further studies showed that the cell cycle was stalled in the G1, and incomplete mitotic cell division was observed. Meanwhile, obacunone activated caspase-3/-7 and induced apoptosis. Obacunone treatment also induced cellular aneuploidy, increasing the number of aneuploid SH-SY5Y cells, which is used as a marker to predict neuroblastoma aggressiveness and response to chemotherapy [18].

##### Effects on Adrenocortical Tumor Cells

High concentration obacunone (≥80 μM) exhibited a toxic effect on mouse adrenocortical tumor Y1 cells, which significantly increased the number of cells in the G1 phase and enhanced mitochondrial membrane brightness; the expression of MFN1 and MFN2 proteins drastically declined, in addition to the expression of corticosterone synthesis-related enzymes and transcription factor-regulating enzymes, thus inhibiting corticosterone synthesis in adrenal cortical cells [29].

##### Effects on Non-Small Cell Lung Cancer (NSCLC) and Melanoma Cells

Previous studies have shown that obacunone is cytotoxic to human NSCLC A549 cells (IC_50_ = 25 μM [20,30]) and melanoma SK-MEL-2 cells (IC_50_ = 19.71 μg/mL) [20].

##### Other Effects

To a certain extent, obacunone had cytotoxicity on ovarian cancer cell SKOV3 (IC_50_ > 60 μM), skin melanoma cell SKMEL2 (IC_50_ = 43 μM), central nervous system cell XF498 (IC_50_ > 60 μM) [20], leukemia cell lines CCRF-CEM cells (IC_50_ = 33.77 ± 5.46 μM), leukemia cell lines multidrug-resistant P-gp-overexpressing subline CEM/ADR5000 cells (IC_50_ = 28.99 ± 3.18 μM), U87MG glioblastoma cells (IC_50_ = 38.47.99 ± 3.20 μM), and EGFR-transfected U87MG.ΔEGFR subline (IC_50_ = 49.22 ± 2.98 μM) [6]. In addition, like verapamil, obacunone had the ability to inhibit p-gp activity. Obacunone (40 μM) could increase the toxicity of vincristine to mouse leukemic lymphocytes L1210 cells by approximately 10-fold (vincristine EC_50_ = 0.9 μM), and colchicine (colchicine EC_50_ = 1.3 nM) was three times more toxic when combined with obacunone [28]. Interestingly, obacunone was limited to enhancing the effects of microtubule inhibitors without significant cytotoxicity potentiation with other antitumor agents, such as adriamycin, cisplatin, or 5-fluorouracil [28].

The combination of obacunone (40 μM) with vincristine increased the toxicity of vincristine to mouse leukemic lymphocytes L1210 cells by approximately 10-fold (vincristine EC_50_ = 0.9 μM), colchicine (colchicine EC_50_ = 1.3 nM) was three times more toxic when combined with obacunone. Interestingly, obacunone was limited to enhancing the effects of microtubule inhibitors without significant cytotoxicity potentiation with other antitumor agents, such as adriamycin, cisplatin, or 5-fluorouracil [28]. 

Previous studies concluded that low concentrations of obacunone did not induce cytotoxicity in cells derived from normal tissues or affect cell activity through observations of the cell number and morphology [6,18,19,24,31,32,33,34,35,36]. Cell viability was slightly reduced at high concentrations (1280 μM); however, no cytotoxicity was observed [32]. In contrast, obacunone is more cytotoxic to cancer cells, with a dose-dependent induction of apoptosis or inhibition of proliferation. Table 2 summarizes the impacts of obacunone on normal cells; the main mechanisms underlying the anticancer effect of obacunone on cancer cells in vitro are presented in Table 3.

### 3.2. Anti-Inflammatory Effects

Obacunone exerts anti-inflammatory effects [37]. Obacunone at 25–100 mg/kg/d administered by gavage all effectively attenuated the severity of DSS-induced ulcerative colitis in mice by mitigating the Toll-like receptor 4/nuclear factor-κB signaling hyperactivation and regulating the abnormal composition of the intestinal flora [38]. Obacunone in a dose-dependent manner (10–50 mg/kg/d) alleviated experimental autoimmune prostatitis-induced chronic pelvic pain syndrome and pro-inflammatory depolarization of macrophages within the prostate via, at least partially, deactivating macrophage migration inhibitory factor (MIF) [39]. 

Cellular experiments showed that obacunone (at 100–400 μM) could inhibit MIF activity by docking with the position between the MIF pocket A and C chains, increasing mitogen-activated protein kinase (MAPK) phosphatase-1 (MPK-1) expression, which in turn promoted p-p38 MAPK dephosphorylation, thereby reducing the expression of AP-1 downstream pro-inflammatory factors, such as interleukin (IL)-Iβ, monocyte chemoattractant protein-1, and IL-6 mRNA [22,38,40]. However, obacunone did not act on the CpG-binding protein (CXXC) motif-mediated thiol-protein oxidoreductase activity of MIF [40], thus inhibiting the MIF-induced pro-inflammatory response while preserving the beneficial effects of MIF on cellular redox homeostasisa favorable effect [40]. 

In addition, obacunone reduced nitric oxide (NO) production by inhibiting the inducible nitric oxide synthase [23,24] (IC_50_ = 11.3 ± 1.5 μM), thus ameliorating neuroinflammation in lipopolysaccharide (LPS)-induced neuroinflammation in mouse BV-2 microglial cells [37]. A recent computer molecular docking study showed that among the 403 compounds screened, only obacunone passed all tests, including evaluating the pharmacokinetic characteristics, toxicity, and binding performance with nitric oxide synthase 3 (NOS3). Obacunone and limonin could bound to the same active site residue Trp447 through different types of bonds (pi–pi stacked and H-bonds, respectively), creating a more stable protein structure to assist in maintaining the position of limonin within NOS3, suggesting the synergistic effects against NOS3 associated with hyperuricemia between limonin and obacunone [12]. 

### 3.3. Antifibrosis Effects

In a CCl_4_-induced liver fibrosis model, obacunone (1.5, 3, and 6 mg/kg) decreased alanine aminotransferase/aspartate aminotransferase (ALT/AST) levels and reversed the pathological changes in the liver tissue, with reduced expression levels of epithelial-mesenchymal transition-related proteins (e.g., α-smooth muscle actin and connective tissue growth factor) and lipid oxidation factors and enhanced expression of glutathione peroxidase 4 Gene (GPx-4). The administration of obacunone also inhibited pulmonary fibrosis in bleomycin-induced mice [41] and bile-duct ligation-induced cholestatic fibrogenesis mice [42]. Mechanistic studies revealed that the antifibrotic effect was not only related to anti-inflammatory and antioxidant properties of obacunone but also its regulation of the expression and production of GPx-4, the inhibition of TGF-β/SMAD signaling pathway [43,44], and the maintenance of the homeostasis of bile acids. A recent study has shown that obacunone (10 and 40 mg/kg, intraperitoneal injection) can inhibit ferroptosis by activating the nuclear factor erythroid 2-related factor 2 (NRF2)/GPx4 signaling pathway, thereby improving renal interstitial fibrosis in unilateral ureteral obstruction model mice [45].

### 3.4. Antioxidative Stress Activity

At 40 μM, obacunone significantly protected against oxidative damage caused by high glucose in NRK-52E cells. Obacunone exerted protective effects by causing an increase in antioxidant (such as superoxide dismutase [SOD], glutathione [GSH], and catalase) levels, inhibiting ROS production, and stabilizing mitochondrial membrane potential. It (20–80 μM) also significantly downregulated the glycogen synthase kinase-3β (GSK-3β, NRF2 negative regulator [46]) activity and enhanced nuclear translocation and mRNA expression of NRF2 [32].

Ultraviolet radiation (UVR) causes severe oxidative damage in the retinal pigment epithelial (RPE) cells. Obacunone (2.5 mg/kg) injected into the vitreous cavity of mice inhibited light-induced retinal damage. Obacunone (25 μM) activated the NRF2 signaling cascade response in ARPE-19 cells and primary mouse RPE cells, which promoted the transcription and expression of antioxidant response element-dependent genes, relieved UVR-induced RPE cytotoxicity, and inhibited ROS accumulation, mitochondrial depolarization, lipid peroxidation, and single-stranded DNA accumulation, thereby reducing UVR-caused RPE cell apoptosis [36]. Obacunone treatment at 25 and 50 µM attenuated oxidative stress, sunburn reaction, and photocarcinogenesis in both keratinocytes and full-thickness skin models exposed to solar-simulated radiation [47].

Acute lung injury (ALI) is a life-threatening respiratory failure caused by endogenous or exogenous pathogenic factors [48]. Intraperitoneal injections with obacunone (2.5, 5, and 10 mg/kg) effectively alleviated LPS-induced ALI in mice by activating NRF2 and inhibiting NRF2 ubiquitinated proteasome degradation. Obacunone also reduced LPS-induced ALI and inflammation by inhibiting ferroptosis and the pathological processes of pneumonia and pulmonary edema. Interestingly, treatment with obacunone (10 mg/kg) and Ferrostatin-1 (Fer-1, 5 mg/kg, ferroptosis inhibitor) showed similar effects on LPS-induced ferroptosis and inflammation mouse models of ALI. In cell experiments, obacunone (10–40 µM) inhibited lactate dehydrogenase release and levels of IL-1β, IL-6, and tumor necrosis factor-α in BEAS-2B cells [49].

Central nervous system neurons are highly susceptible to oxidative stress [50], which induces neuronal cell death [51]. Oxidative stress mediates glutamate-induced excitotoxicity [52]. Obacunone significantly inhibited the increase of Ca^2+^ in glutamate-injured cortical cells with an EC_50_ value of 0.039 ± 0.004 μM, restored the mitochondrial membrane potential of glutamate-injured cells to 80% of that of control cells, and reduced glutamate-induced overproduction of NO and peroxides [53]. Meanwhile, obacunone (0.05/0.1 μM) improved the cellular antioxidant defense system and reduced glutamate-induced neurotoxicity by preserving the glutamate-depleted GSH content while restoring glutamate-reduced SOD, glutathione reductase, and GPx activity [53]. Experiments in mouse hippocampal HT22 cells showed that obacunone (25–150 μM) increased p38 MAPK phosphorylation through the p38 MAPK pathway and induced the expression of heme oxygenase-1 (HO-1) [49,54,55], which is regulated by the p38 MAPK pathway to protect neuronal and non-neuronal cells from oxidative stress [56].

**Figure 2:**
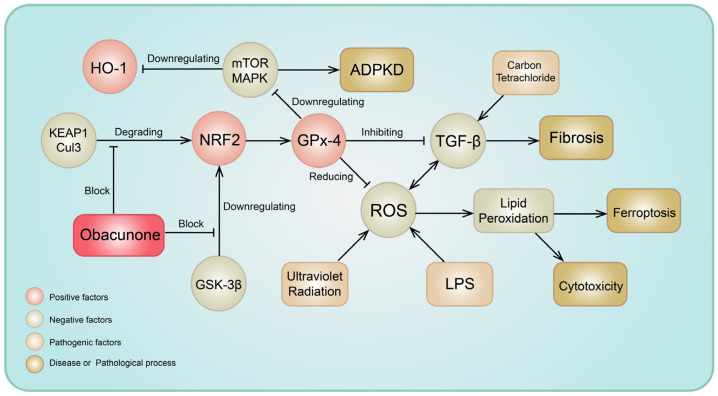
Mechanisms involved in the interactions between obacunone and NRF2.

NRF2 is a critical transcription factor in cellular defense against oxidative stress responses and is present in almost all human body cells. As an effective NRF2 agonist, the summary of the effects of obacunone on the metabolic network through nrf2 is shown in Figure 2.

### 3.5. Antimicrobial Effects

#### 3.5.1. Antibacterial Effects

##### Effects on *Escherichia coli* and *Vibrio harveyi*

Quorum sensing (QS) is a mechanism of communication between microorganisms that allows bacteria to express different physiological behaviors, including virulence of pathogenic microorganisms, antibiotic resistance, biofilm formation, and growth [57]. Biofilms can provide a site for bacterial aggregation and avoid the spread of population QS molecules [58]. Among the five bioactive limonoids studied, namely, obacunone, limonin, nomilin, deacetyl nomilin, and limonin 17-β-D-glucopyranoside, obacunone can most effectively inhibit the cell-cell communication activity mediated by two autoinducers, namely, *N*-acyl homoserine lactone (IC_75_ = 42.66 μM) and Autoinducer-2 (IC_75_ = 28.18 μM), in a concentration-dependent manner (appropriate amount of dimethyl sulfoxide (DMSO) used as the control to eliminate solvent effects). Obacunone inhibits population-sensing-dependent virulence (e.g., Shiga toxin type 2), flagellar and motility gene expression, and biofilm formation by the intestinal pathogens *E. coli* O157:H7 (EHEC) (IC_50_ = 116.68 μM) and *V. harveyi* (a gram-negative marine bacterium [57]) (IC_50_ = 91.2 μM) to exert antibacterial effects [25,59]. 

##### Effects on *Staphylococcus aureus*

*Staphylococcus aureus* (SAU) is the prevailing pathogen in post-traumatic infections, with the emergence of antibiotic resistance presenting formidable treatment hurdles. Obacunone, one of the two active ingredients of ShangKeHuangShui, a patented traditional Chinese herbal formula used in averting post-traumatic infections, can bind to protein tyrosine phosphatase PtpA (ptpA) of SAU with the binding energy of −8.3 kcal/mol as shown in ptpA docking screening. This discovery laid the foundation for the potential application of obacunone in treating SAU infection [7].

##### Effects on *Salmonella typhimurium* LT2

*Salmonella* is an important commensal pathogen and a priority surveillance target for public health worldwide. The pathogenicity of *Salmonella* is mainly associated with specific regions of pathogenicity islands (SPIs, also called pathogenicity islands) encoding pathogenesis-related genes distributed in clusters on chromosomes [60]. SPI1 and SPI2 are closely associated with *Salmonella* pathogenicity; hilA is a critical regulatory protein of SPI1 [61]. SPI1 and SPI2 encode a different type III secretion system (TTSS), which acts as a molecular injector to inject virulence and effector proteins directly into host cells, affecting cellular function and promoting infection [62]. Obacunone (6.25–100 μg/mL, appropriate amount of DMSO used as the control to eliminate solvent effects) can dose-dependently inhibit hilA in an EnvZ-dependent fashion and thus affect the expression and function of the TTSS, thereby reducing *Salmonella* virulence [59,62]. Notably, the carbon number of A-ring and the double bond between C1 and C2 allow obacunone to have the best antagonistic activity against TTSS compared with nomilin and deacetylnomilin, the other two common limonin compounds. Obacunone (100 μg/mL) also downregulated the levels of maltose and maltose transporters that promote bacterial uptake and efficient catabolic metabolism, resulting in reduced maltose uptake by *Salmonella*. Meanwhile, obacunone (100 μg/mL) inhibited three hydrogenase manipulators involved in *Salmonella* metabolism [63]; the inhibition of hydrogenase and SPIs may also have a cumulatively enhanced virulence-reducing impact [62]. Thus, it may serve as a lead compound for developing antibacterial strategies against *S. typhimurium*.

##### Effects on *Vibrio parahaemolyticus*

Fibrillar adhesins are proteins with immunoglobulin-like fold(s) involved in biofilm formation and cell-cell interactions. In a computer simulation study, four novel fibrillar adhesin-like proteins, WP_005477759.1, WP_005480168.1, WP_005489282.1, and WP_005490731.1, were identified in *Vibrio parahaemolyticus*. A previous study analyzed the binding of 277 compounds to these four proteins and found that, except for the slightly weaker binding of WP_005480168.1, the binding of obacunone to the remaining three ranked in the top five, revealing its potential to inhibit the virulence of *Vibrio parahaemolyticus* on the host [64].

#### 3.5.2. Antifungal Activity

Obacunone showed significant antifungal effects against *Candida albicans* in in vitro drug sensitivity tests and a murine model of disseminated candidiasis, and, at 12.5–100 μg/mL, inhibited the growth of *C. albicans* in a concentration-dependent manner, with concomitant shortening of mycelial length in vitro. In a murine model of systemic disseminated candidiasis, obacunone (5 mg/kg) administration significantly prolonged the lifespan of infected mice and enhanced resistance to disseminated candidiasis. The mean survival time of the model mice was similar to that of mice in the fluconazole-administered group. Since the main virulence factor of *C. albicans* is the production of mycelia in the form of bacilli, it has been hypothesized that the antifungal effect of obacunone is mediated by its inhibition of mycelium production [65]. 

However, obacunone is not effective against many fungi. Earlier experiments have demonstrated that obacunone (100 μg/mL) does not affect the growth inhibition of *Cladosporium cucumerinum* [66]. 

#### 3.5.3. Potential Antiviral Activity

In a drug screening experiment for five possible protein targets, namely, 3C-like protease (3CL^pro^), papain-like protease (PL^pro^), RNA replicase (RdRp), spike glycoprotein receptor-binding domain (SpG-RBD), and angiotensin-converting enzyme 2 (ACE2) in severe acute respiratory syndrome coronavirus 2 (SARS-CoV-2), investigators showed that obacunone might have potential activity against SARS-CoV-2, based on molecular docking and absorption, distribution, metabolism, excretion, and toxicity properties [67,68,69,70]. Obacunone could interact with the significant protease 3CL^pro^ catalytic dimer, PL^pro^ catalytic triad, and RdRp active site and bind to SARS-CoV-2 at the catalytic site of ACE2 and/or the RBD site [71]. Additional studies have shown that obacunone forms two hydrogen bonds with Tyr453 and Arg403 residues and two other hydrocarbon bonds with Tyr495 and Gln498 residues [67]. The density functional theory docking and molecular dynamics simulation study revealed that obacunone has a highly reactive nature and a stable binding interaction with a possibly high biological interaction [72]. Unlike remdesivir and dexamethasone, obacunone forms two bonds with Arg495 and Gln401 in the active site of the spike protein, with lower binding energy [68]. Notably, Magurano et al. confirmed the in vitro virucidal activity of obacunone against a SARS-CoV-2 viral isolate obtained from a patient with COVID-19 at an IC_50_ of 31 μg/mL [73]. 

### 3.6. Endocrine and Metabolic Effects

#### 3.6.1. Anti-Obesity Effects

Activation of G protein-coupled bile acid receptor 1 (also known as Takeda G protein-coupled receptor 5 [TGR5]), a member of the G protein-coupled receptor superfamily, enhances the release of glucagon-like peptide-1 (GLP-1) [74], thereby reducing serum glucose levels and improving glucose tolerance [75,76]. Previous studies have demonstrated that obacunone (1–100 μM) can transcriptionally activate TGR5 and enhance the TGR5-GLP-1 pathway in a dose-dependent manner [77], thus inhibiting adipocyte differentiation in 3T3-L1 cells. After administration of 0.1% obacunone, diabetic KK.Cg-A^y^ mice showed increased GLP-1 secretion and significantly reduced visceral and subcutaneous fat accumulation, obesity, and hyperglycemia [76]. In addition, obacunone stimulated a significant increase in quadriceps and gastrocnemius muscle weights in KK.Cg-A^y^ mice, similar to the results of skeletal muscle development and hypertrophy observed in other studies in which mTOR was activated [78]. The activation effect of obacunone on TGR5 has also been confirmed in another study; mice fed a high-fat diet supplemented with obacunone had lower body weights and blood glucose levels as well as enhanced glucose tolerance. Limonin, the most abundant limonoid in citrus seeds, shares a similar structure to obacunone and does not affect TGR5 owing to the A-ring blocking, unlike in obacunone [1,77]. In addition, obacunone has a stronger inhibitory effect on α-glucosidase than the positive drug acarbose, which also explains its hypoglycemic effect [79].

Peroxisome proliferator-activated receptor γ (PPARγ) is a member of the nuclear hormone receptor superfamily involved in regulating a complex transcriptional network associated with lipid metabolism and glucose homeostasis [80]. Obacunone antagonizes PPARγ activity, thereby inhibiting lipid accumulation during adipocyte differentiation [76].

#### 3.6.2. Regulation of Cholesterol Metabolism

Sterol regulatory element-binding proteins (SREBPs) are crucial to cholesterol control in eukaryotic cells [81,82]. When cells are deficient in cholesterol, SREBPs bind to the SREBP cleavage-activating protein (SCAP) and move from the endoplasmic reticulum to the Golgi apparatus where they undergo proteolytic cleavage and subsequently translocate to the nucleus and activate transcription of target genes for the low-density lipoprotein receptor or HMG-CoA synthase to increase cholesterol levels [83]. When cellular cholesterol levels are high, SCAP senses cholesterol and undergoes conformational changes, binds to insulin-induced gene (INSIG) proteins [84], and prevents the movement of the SCREP-SCAP complex from the endoplasmic reticulum to the Golgi apparatus [35]. Kim et al. showed that obacunone (10–100 μM), which has a cholesterol-like tetracyclic structure, can induce a conformational change in SCAP, making it insensitive to cholesterol and inhibiting its binding to INSIG in the presence of intracellular cholesterol. Notably, the presence of obacunone allowed the SREBP-1/SCAP complex to move to the Golgi apparatus for SREBP-1 processing and activated SREBP1 cleavage. As a result, the total level of the SREBP-1 protein increased concentration-dependently with obacunone, with a significantly higher ratio of mature to precursor forms, which upregulated the expression levels of genes associated with cholesterol and lipid metabolism [35].

### 3.7. Effects on Bones Metabolism

RUNT-related transcription factor 2 (RUNX2) is a crucial transcription factor involved in osteoblast differentiation [85,86]. The bone morphogenetic protein 2 (BMP2) and β-catenin pathways upregulate *RUNX2* expression [87], which increases the transcriptional activity of RUNX2 [88], whereas GSK3 degrades β-catenin and inhibits the transcriptional activity of RUNX2 [88]. Obacunone (1 and 10 μM) promotes early and late osteoblast differentiation by increasing the expression of BMP2, phosphorylation of smad1/5/8 and β-catenin, and inhibition of GSK3 [31]. Another study demonstrated the therapeutic effects of obacunone in bone loss model mice induced by ovariectomy [89,90] via downregulating the expression of integrin α1, attenuating the activation of focal adhesion kinase (FAK) and steroid receptor coactivator (Src) signaling, and targeting MIF to impede osteoclastogenesis. These findings suggest that obacunone is a therapeutic candidate to treat or prevent bone diseases, such as osteoporosis.

### 3.8. Effects on Arginase and Ferroptosis

Vascular endothelial arginase reduces endothelial nitric oxide synthase (eNOS) activity by depleting l-arginine [91], thereby decreasing NO concentrations and leading to vascular diseases. Therefore, arginase inhibitors are a potential strategy for treating atherosclerotic vascular disease [92]. While screening arginase inhibitors, obacunone (2–32 μM) inhibited arginase activity and increased NO production by enhancing the stability of eNOS dimers [93,94]. In wild-type (WT) and atherogenic mouse models (ApoE^−/−^) fed a high-cholesterol diet, obacunone (30 μM) restored vasodilation to WT levels within 18 h. These findings suggest that obacunone can prevent or treat vascular diseases induced by endothelial dysfunction [94].

Myocardial ischemia/reperfusion injury (MIRI) is a common challenge in reperfusion therapy for myocardial infarction. Ferroptosis is a novel form of programmed cell death that relies on iron, and iron deposition and ROS levels are important regulatory factors for iron death. Obacunone (1.5 and 6 mg/kg for 7 days via intraperitoneal injection) can activate the Nrf2 signaling pathway, reducing oxidative stress levels, inhibiting ferroptosis, and thereby improving myocardial injury in MIRI rats [95].

### 3.9. Other Effects: Insect-Repellent Effects, Insecticidal Effects, and Shortening of Sleep Time in Mice

Obacunone inhibits mosquito (*Culex quinquefasciatus*) molting (EC_50_ = 6.31 ppm, EC_95_ = 44.67 ppm) [96] and induces food refusal in Colorado potato beetle larvae (100/31.7/10/3.2 μg/disk) [97,98]. Dibromo-obacunone, prepared by selective bromination and palladium-catalyzed coupling, exhibited higher insecticidal activity than obacunone and toosendanin [99].

In addition, obacunone (0.1% with food) shortened the duration of sleep induced by the transabdominal administration of α-chloralose (50 mg/kg) and urethane (100 mg/kg) in mice [100].

## 4. Pharmacokinetic Studies of Obacunone

Following administration at 10 mg/kg via gavage, obacunone was rapidly absorbed into the blood, reaching a maximum plasma concentration of 202.75 ± 36.11 ng/mL with a T_max_ of 1–2 h, with a plasma AUC of 591.59 ± 109.41 ng · h/m and oral bioavailability of approximately 14% [101]. The pharmacokinetic data of obacunone are presented in Table 4.

**Figure 3:**
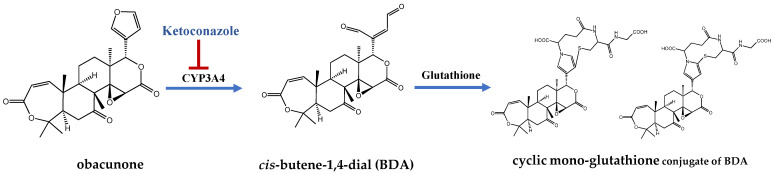
The main metabolic process of Obacunone.

The co-incubation of obacunone with human liver microsomes (LMs) showed that the reduction, hydroxylation, and glycation reactions at the C-7 and C-16 sites are the main metabolic pathways of obacunone [103]. In the NADPH-added microsomal incubation system, obacunone was first metabolized to *cis*-butene-1,4-dial (BDA), which was then captured by glutathione, *N*-acetylcysteine, and *N*-acetyllysine to generate 13 metabolites. Among these metabolites, the cyclic mono-glutathione conjugate of BDA was the predominant metabolite, which was also detected in the bile and urine after obacunone administration in rats (10 mg/kg, i.v.). The generation pathway of the main metabolites is illustrated in Figure 3. The intermolecular reactions of GSH and BDA intermediates of the adducts generated four bis-GSH-BDA adducts, which could be found in rat bile. In addition, obacunone can be NADPH-independent and directly pass through the Michael receptor (*α*,*β*-unsaturated carbonyl group) without activation to obtain obacunone-derived GSH adducts excreted through bile with GSH, which can further adduct with acetylcysteine (NAC) to form NAC conjugates excreted in the urine [104]. Recombinant enzyme and ketoconazole inhibition experiment showed that CYP3A4 is the key enzyme responsible for the metabolic conversion of obacunone. 

Notably, there are different metabolic processes of obacunone in LMs (including humans, monkeys, dogs, rats, and mice) and zebrafish. The product obtained following C-7 hydrogenation was the primary metabolite of obacunone in LMs and zebrafish. In contrast, the C-16 hydrogenation product was detected only in LMs, suggesting species differences in the metabolism of obacunone [105]. 

## 5. Safety Profile of Obacunone

Obacunone is a potential hepatotoxic component [101]. Lang et al. showed that the oral administration of 50 mg/kg of obacunone significantly increased serum ALT and AST levels in mice [104]. Previous studies have shown that BDA is derived from the furan ring, indicating that it is highly reactive, and cellular nucleophiles such as proteins, RNA, or DNA can react with it, thus inducing toxicity [106]. Obacunone is activated by CYP3A4 in vivo, and the opening of the furan ring to form the reactive intermediate BDA may be responsible for the hepatic damage [104]. Other studies have reported that feeding rats a diet supplemented with 0.05% obacunone for 38 days did not result in slowed body weight gain, and no pathological changes in the tissues and organs were observed [14,15,62]. Therefore, an in-depth study of the relationship between the toxicity of obacunone in animals and its dose remains warranted.

## 6. Outlook

**Figure 4:**
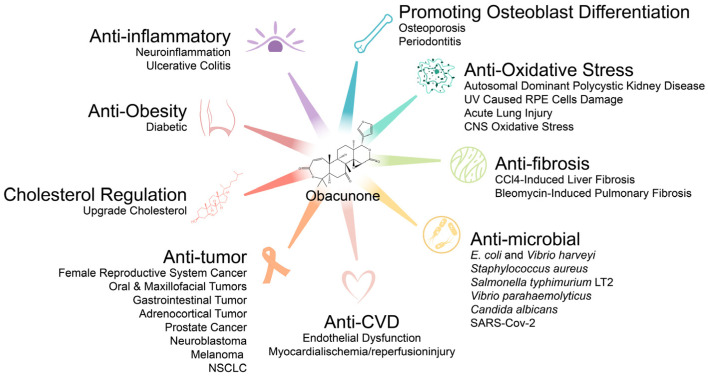
Summary of obacunone biological activities.

In the extensive pharmacological effects of obacunone (Figure 4), it is important to highlight that the antitumor effects of obacunone have attracted significant attention owing to its strong cytotoxicity against tumor cells, but not normal cells and ability to reverse P-gp-induced multidrug resistance in drug-resistant tumors. In addition, the antimicrobial activity of obacunone should also be considered, especially its effect on COVID-19, since drug treatments against COVID-19 are lacking. As existing studies primarily focused on in vitro cellular experiments, certain in vivo pharmacological effects and underlying mechanisms have not been fully elucidated, such as its effects on cancer, infections with pathogenic microorganisms, and metabolic diseases and bioavailability of obacunone following oral administration; therefore, future research should focus on elucidating the mechanism(s) of action of obacunone, potential clinical applications, and structural modifications or development to improve bioavailability. 

## 7. Conclusions

This review offered a comprehensive summary of obacunone biological activities, including antitumor, anti-inflammatory, antioxidative stress, antifibrosis, and antimicrobial effects and explained its effect on the endocrine, bone, and cardiovascular system and potential mechanisms, proposing new opportunities for the utilization of obacunone. Considerable progress in unraveling the mechanisms of action of obacunone in various diseases has been found, such as the inhibition of the TGF-β/SMAD signaling, MIF/RANKL/MAPK/NF-κB, TLR4/NF-kappaB signaling cascade, Nrf2/GPx4 signaling, and TGR5/GLP-1 and PPAR gamma pathways. This will promote the development and application of obacunone in clinical settings.

## Figures and Tables

**Table 1:** Traditional Chinese Medicine with obacunone as a core ingredient.

Herbs and Traditional Chinese Medicine Formula		Function of the Traditional Chinese Medicine	Role
Herb	*Dictamnus dasycarpus Turcz.* (Chinese name: Bai xian pi)	Clearing heat, drying dampness, dispelling wind, and detoxification.	The qualitative and quantitative indicator components [2].
	*Phellodendron chinense Schneid.* (Chinese name: Huang bo)	Clearing heat, drying dampness, purging fire, removing steam, detoxifying, and treating sores.	The qualitative indicator component [2].
	*Coptis chinensis Franch.* (Chinese name: Huang lian)	Clearing heat, drying dampness, purging fire, and detoxifying.	The key phytochemical component against ulcerative colitis [3].
	*Euodia rutaecarpa* (*Juss.*) *Benth.* (Chinese name: Wu zhu yu)	Dispelling cold, relieving pain, lowering reversal, stopping nausea, assisting yang, and stopping diarrhea.	The highly active compound for liver cancer [4].
	*Lippia javanica* (*Burm. F.*) (Farecha)	Treatment of fever, malaria, cough, cold, chest pain, asthma, bronchitis, and diarrhea.	The key phytochemical component with antioxidant and liver protection properties [5].
	*Araliopsis soyauxii Engl.* (*Rutaceae*)	Treatment of lung diseases, malaria, and gonorrhea.	The main active component for antitumor activity [6].
Traditional Chinese Medicine Formula	ShangKeHuangShui, composed of Coptis chinensis Franch. (Huang lian), *Phellodendron chinense Schneid.* (Huang bo), *Gardenia jasminoides Ellis* (Zhi zi), *Arnebia euchroma* (*Royle*) *Johnst.* (Zi Cao), *Arnebia euchroma* (*Royle*) *Johnst.*(Bo he), and Ming fan	Treatment of staphylococcus aureus infection.	The active chemical component against bacterial infection [7].
	Erchen decoction, composed of *Atractylodes macrocephala Koidz.* (Bai zhu), *Citrus reticulata Blanco* (Ju hong), *Poria cocos* (*Schw.*) *Wolf* (Fu ling), *Glycyrrhiza uralensis Fisch.* (Gan cao)	Regulation of multiple core target genes in the pathogenesis of atherosclerosis.	The core component for the treatment of atherosclerosis [8].
	Chaihu Shugan San, composed of Bupleurum chinense DC. (Chai hu), *Citrus aurantium* L. (Zhi qiao), *Ligusticum chuanxiong Hort.* (Chuan xiong), *Aconitum carmichaelii Debx.* (Fu zi), *Paeonia lactiflora Pall.* (Bai shao), *Curcuma longa* L. (Yu jin), *Lygodium japonicum (Thunb.) Sw.* (Hai jin sha)	Prevention of cholesterol gallstone formation.	The critical active metabolite in regulating bile acid metabolism [9].
	Huazhuo Quyu Formula, composed of Rheum palmatum L. (Da huang), *Coptis chinensis Franch.* (Huang lian), *Salvia miltiorrhiza Bge.* (Dan shen), *Curcuma phaeocaulis VaL.* (E zhu), *Curcuma Longa* L. (Jiang huang), *Lysimachia christinae Hance* (Jin qian cao)	Treatment of Wilson’s Disease-associated liver fibrosis.	The essential active compound that inhibited liver inflammatory processes and vascular hyperplasia regulated the cell cycle and suppressed both the activation and proliferation of hepatic stellate cells [10].
	Huanglian Jiedu decoction, composed of Coptis chinensis Franch. (Huang lian), *Scutellaria baicalensis Georgi* (Huang qin), *Phellodendron chinense Schneid.* (Huang bo), *Gardenia jasminoides Ellis* (Zhi zi)	Treating acute lung injury.	The active compound inhibits inflammasome activation via the sphingolipid pathway [11].
	Simiao pill, composed of *Atractylodes lancea* (*Thunb.*) *DC.* (Cang zhu), *Achyranthes bidentata Bl.* (Niu xi)*, Phellodendron chinense Schneid.* (Huang bo)*, Coix lacryma-jobi L.var.ma-yuen (Roman.) Stapf* (Yi yi ren)	Treating hyperuricemia.	The synergistic effects of limonin against nitric oxide synthase 3 [12].

**Table 2:** Impacts of obacunone on the normal cell lines.

Species		Cell Line	Dose Used (μM)	Impacts
Mouse	Normal Embryo Fibroblast	NIH/3T3 cells [19]	6.25–200	No cytotoxicity
Mouse	Normal Bone	MC3T3-E1 cells [31]	1–100	No cytotoxicity
Mouse	Normal Liver	AML12 cells [6]	IC_50_ > 100	No cytotoxicity
Rat	Normal Kidney	NRK-52E cells [32]	40–1280	No cytotoxicity
Cricetulus	Normal Ovary	Chinese hamster ovary cells [18]	10–40	No cytotoxicity
Canine	Normal Kidney	MDCK cells [33]	0.78–50	No cytotoxicity
Human	Normal Prostate	RWPE-1 cells [24]	6.25–200	No cytotoxicity
Human	Normal Breast	MCF-12F cells [34]	6.25–200	No cytotoxicity
Human	Normal Epidermis	HaCaT cells [35]	25–100	No cytotoxicity
Human	Normal Retina	ARPE-19 cells [36]	1–50	No cytotoxicity

**Table 3:** Effect of obacunone on the cancer cell lines.

Types of Cancer Cell Lines		Effect	Mechanism	Reference
Colon cancer	Caco-2 cells	An apparent dose–response antiproliferative effect was observed throughout 24–48 h incubation at 10–50 μM concentration.	Obacunone arrested the cell cycle process, accumulating cells in the G1 and G2 phases.	[18]
	SW480 cells	The IC_50_ values of obacunone were 97.02 ± 4.1 μM and 56.22 ± 4.03 μM for 24 h and 72 h, respectively.	Obacunone induced apoptosis by activating the intrinsic apoptosis pathway and activating p21, leading to cell arrest at the G2/M phase of the cell cycle.	[19]
Liver cancer	HepG2 cells	The IC_50_ values of obacunone on cell number, nuclear intensity, cell membrane permeability, and concentration of reactive oxygen species were 42.87 μM, 54.09 μM, 84.00 μM, and 41.51 μM for 48 h incubation, respectively.	The potential mechanism of hepatotoxicity might be associated with changes in the cell number, nuclear intensity, cell membrane permeability, and concentration of reactive oxygen species, which may induce cell apoptosis.	[21]
Pancreatic cancer	Panc-28 cells	Obacunone demonstrated both time (2, 4, and 6 days) and dose-dependent (50 μM and 100 μM) inhibition of cell proliferation.	The cytotoxicity was associated with tumor suppressor protein (p53) activation and proapoptotic and anti-inflammatory pathways.	[22]
Prostate cancer	LNCaP cells	Obacunone had a time- and dose-dependent inhibition of cell proliferation, with more than 60% inhibition of cell viability at 100 μM after 24 and 48 h of incubation.	Obacunone caused cytotoxicity to cells by activating intrinsic apoptosis, suppressing inflammation, and down-regulating androgen receptors and prostate-specific antigens.	[24]
	22RV1 cells	Obacunone at the concentrations of 21.25 μM, 42.5 μM, and 85 μM showed intense inhibitory ability, with the highest inhibition rate of 78.9% at 85 μM. The proportion of apoptosis cells was raised progressively in a time-dependent mode after 24 h, 48 h, and 72 h incubation at concentrations of 85 μM of obacunone treatment.	Obacunone effectually controlled proliferation and promoted apoptosis in 22RV1 prostate cancer cells, which were related to twenty-one proposed metabolites, and nicotinate and nicotinamide metabolism, phenylalanine metabolism, tryptophan metabolism, as well as ascorbate metabolism.	[25]
Breast cancer	MCF-7 and MDA-MB-231 cell	(1) Obacunone (200 μM, 72 h) exhibited the cytotoxicity of 44% and 18% of MCF-7 and MDA-MB-231 cells, respectively. (2) Obacunone (20 μM, 30 min) exhibited potent aromatase inhibition activity with an IC_50_ value of 28.04 μM. (3) Caspase-7 in MCF-7 cells treated with obacunone (200 μM, 72 h) was altered 3.6-fold.	The antiproliferative properties of obacunone were mediated by caspase-7-dependent pathways.	[6,34]
Neuroblastoma	SH-SY5Y cells	(1) An apparent dose–response antiproliferative effect was observed throughout 24–48 h at the concentration of 10–50 μM obacunone (25 μM, 36 h). (2) Obacunone (25 μM, 36 h) could induce the caspase 3/7 activity and aneuploidy.	The mechanism of obacunone action was related to apoptosis induction, cell cycle arrest, and aneuploidy.	[18]
Adrenocortical tumor	Y1 mouse adrenocortical tumor cells	(1) The inhibitory rate of obacunone (2.5–40 μM for 24 h) on cell growth was about 35%, while 160 μM had a cell inhibition rate of over 60%. (2) The backbone (more than 80 μM for 24 h) had cytotoxicity.	Obacunone inhibited corticosterone synthesis by corticosterone in adrenal cortex cells, which might be related to cell cycle arrest and the expression of steroid synthase on the mitochondrial membrane.	[29]

**Table 4:** Detailed pharmacokinetic data of obacunone.

Drug Components	Dosage	Route of Administration	Animal Model	Obacunone Pk/Pd Parameters	Ref.
Obacunone	10 mg/kg	Oral	Rat	AUC_0-last_ = 804.21 ± 163.73 ng·h/mL C_max_ = 202.75 ± 36.11 ng/mL T_max_ = 1–2 h T_1/2_ = 3.47 ± 0.55 h Vd = 64.68 ± 20.96 L/kg CL = 12.75 ± 2.59 L/kg/h F = 13.59%	[101]
Obacunone	1 mg/kg	Intravenous	Rat	AUC_0-last_ = 591.59 ± 109.41 ng·h/mL C_max_ = 628.52 ± 188.41 ng/mL T_1/2_ = 4.30 ± 1.98 h Vd = 10.79 ± 5.69 L/kg CL = 1.71 ± 0.28 L/kg/h	[101]
*Dictamnus dasycarpus* cortex extract	0.424 g/kg (44.7 mg/kg Obacunone equivalent)	Oral	Rat	AUC_0-last_ = 1701 ng·h/mL C_max_ = 531 ± 56.7 ng/mL T_max_ = 1.00 ± 0.00 h	[102]

Abbreviations: AUC—Area under the curve, C_max_—Maximum serum concentration, T_max_—Time to reach the maximum concentration, T_1/2_—Time required for plasma concentration of a drug to decrease by 50%, Vd—Volume of distribution, CL—Clearance, F—Fraction absorbed.

## Data Availability

No new data were created or analyzed in this study. Data sharing does not apply to this article.
